# Inhalable dust, endotoxins and (1–3)-β-d-glucans as indicators of exposure in waste sorting plant environment

**DOI:** 10.1007/s10453-017-9484-4

**Published:** 2017-05-26

**Authors:** Anna Kozajda, Karolina Jeżak, Marcin Cyprowski, Irena Szadkowska-Stańczyk

**Affiliations:** 1 Nofer Institute of Occupational Medicine, 8 Teresy Str, 91-348 Lodz, Poland; 2 Laboratory of Biohazards, Central Institute for Labour Protection – National Research Institute, Czerniakowska 16, 00-701 Warsaw, Poland

**Keywords:** Occupational exposure assessment, Aerosols, Bioaerosols, Waste recycling, LPS

## Abstract

The aim of the study was to assess the levels of inhalable dust, endotoxins and (1–3)-β-d-glucans as agents harmful to the respiratory tract of workers of municipal waste sorting plants and interaction between these agents based on the measurements taken in two plants with different processing capacities. The study was conducted in summer season in two waste sorting plants (WSPs) differing in processing capacity. Samples of bioaerosol for inhalable dust (gravimetric method), endotoxins (LAL test in kinetic, chromogenic version) and (1–3)-β-d-glucans (Glucatell test in kinetic version) were collected from 42 sorting workers using individual aspirators with glass fiber filters during the work shift. Average geometric concentrations (geometric standard deviation; min–max) of inhalable dust, endotoxins and (1–3)-β-d-glucans were: WSP1: 1.7 mg m^−3^ (2.2; 0.6–6.9 mg m^−3^); 15.9 ng m^−3^ (2.1; 5.4–78.9 ng m^−3^), 55.1 ng m^−3^ (1.8; 20.7–188.6 ng m^−3^) and WSP2: 0.8 mg m^−3^ (2.2; 0.2–3.8 mg m^−3^), 9.8 ng m^−3^ (2.4; 1.6–29.7 ng m^−3^), 45.0 ng m^−3^ (3.2, 5.7–212.9 ng m^−3^), respectively. A significantly higher concentration of inhalable dust was recorded in WSP1 with bigger processing capacity compared to WSP2 (less processing capacity). Significant (*p* < 0.05) and very high correlations (Spearman rank *R* > 0.7) were found between the concentrations of all analyzed harmful agents. Processing capacity of waste sorting plants differentially affects the concentrations of inhalable dust, whereas concentrations of endotoxins and glucans are less clearly affected. This suggests that relative concentrations of endotoxin and glucan are depending on the waste sorting capacity.

## Introduction

Municipal waste sorting plants are specific workplaces because of their workers’ frequent direct contact with the municipal mixed wastes. Waste with a high content of organic fraction during earlier storage in containers undergoes some putrefactive processes depending on climatic conditions (temperature and humidity) (Wouters et al. 2006). Microorganisms growing on organic residue are present at high concentration in the air surrounding the wastes storage in the form of bioaerosol (Douwes et al. 2003; Wouters et al. 2006).

According to Poulsen et al. (1995), workers of recycling plants may be exposed to complex, compound bioaerosols and volatile chemical compounds, where synergistic interactions may occur between particular components. Due to exposure to endotoxins acting similarly to substances which enhance the organism’s immunological response (adjuvants), the organism’s sensitivity is increased, which easily initiates allergic response. Studies on the impact of seasonality on concentration of airborne biological agents indicate that such exposure reaches the highest values in summer (Oppliger et al. 2005; Pinto et al. 2015; Thorn 2001).

Endotoxins are biologically active lipopolysaccharides (LPSs) produced by the most external layer of cellular walls of gram(−) bacteria. Structurally, they are macromolecules with molecular weight from several to a few dozen megadaltons, arising from polymerization of the smaller units of LPS with proteins and phospholipids of the cellular wall; therefore, they are defined as heteropolymers. These compounds become extremely easily released into the external environment through fragmentation of the cellular wall, which bulges and then separates in the form of usually 30–50 nm spherical globules. In organic dusts, the endotoxins occur mainly in this form in the smallest diameter part of the respirable fraction. LPS can accumulate in dust in large quantities even after the death of bacterial cells which produce them (Douwes et al. 2003; Dutkiewicz 2004; Ławniczek-Wałczyk and Górny 2010). LPS inhaled by people activates non-specifically the pulmonary macrophages which secrete many substances exhibiting strong biological effects, defined as mediators of inflammatory reaction (cytokines, biologically active lipids, enzymes, coagulogens, oxygen metabolites). The studies confirmed that the dose–response relationship was observed in occupational exposure to endotoxins (Donham et al. 2000). Inhaled endotoxins may cause bronchial asthma and may exacerbate the course of bronchial asthma, organic dust toxic syndrome (ODTS) and the acute form of byssinosis. People experimentally exposed to endotoxins through inhalation develop fever, chills, joint pain, fatigue and headache (ill-being—influenza-like symptoms), changes in the number of leukocytes in blood, upper airway inflammation, dry cough, chest tightness, decrease in spirometric parameters (including FVC and FEV1) (Douwes et al. 2003; Ławniczek-Wałczyk and Górny 2010). The results of some studies indicate that sensitivity to endotoxins is genetically related and may vary considerably between individuals (Dutkiewicz 2004). Simultaneous exposure to endotoxins and allergens of mold spores in the working environment of those who collect wastes may increase the allergic response in hypersensitive people (Poulsen et al. 1995).

(1–3)-β-d-Glucans are macromolecular polymers of glucose (polysaccharides), which constitute a component of the cellular wall of molds, yeast, certain bacteria as well as plants and algae. These metabolites after cellular decomposition are released into the air and may induce immunosuppressive and inflammatory reactions with symptoms very similar to those observed in exposure to endotoxins. Inflammatory effects of glucans were far less frequently diagnosed and investigated than the similar effects of endotoxins (Douwes 2005; Schleibinger et al. 2004). Inhaled glucans were found to possibly cause an increase in lymphocytes in blood but without any increase in the number of neutrophils and macrophages in the pulmonary secretion observed in the case of exposure to endotoxins (Beijer et al. 2002; Fogelmark et al. 2001; Thorn et al. 2001). Glucans exhibit variable molecular weight and degree of branching of molecules, which may occur in different conformations, including triple helix, single helix or randomly coiled chain, the most prevalent in the environment being the triple helix. Different forms of glucans exhibit different biological activities. Experimental studies on inflammatory reaction in rodents’ respiratory tract indicate that (1–3)-β-d-glucans having a partly open helix have the greatest potential (Foto et al. 2004; Rylander 1999).

The objective of the study was to assess the levels of inhalable dust, endotoxins and (1–3)-β-d-glucans (as harmful agents to the respiratory tract of workers) and correlations between those agents in municipal waste sorting plants environment. The study was conducted based on the indoor air measurements taken at two plants with different processing capacities. These results supplement the rather poor knowledge about occupational exposure of workers in municipal waste sorting plants, which is important in the context of the prospects for high development of this branch and few so far published results of studies on this work environment.

## Materials and methods

### Air sampling strategy

The study was conducted in two municipal waste sorting plants (WSP) of closed type in a big conurbation. The two plants (WSP1 and WSP2) differed in processing capacity (Table 1).

**Table 1:** Characteristics of the studied waste sorting plants

WSP	Efficiency		Average height of the landfill (m)	Time of waste storage (days)	Number of sorting cabins
	Per year (in thousands t)	Per day (t)			
WSP1	82.5	330	3	1	3
WSP2	50.0	170	3	1	1

*WSP* waste sorting plant

Air was sampled in summer in hot weather because such climatic conditions are best for the growth of microorganisms, and consequently the exposure to harmful biological agents in waste management plants is the highest in that season. Analysis of the results did not include workplaces and individual stages of the wastes sorting process, because workers were not assigned to a given activity or workstation and moved all over the hall during the work shift, and exposure was reflected in the mean concentrations of analyzed airborne agents.

The measurement strategy was based on the Polish Standard (PN-EN 13098:2007; PN-91/Z-04030/05). Samples of bioaerosol for analysis of exposure to inhalable dust, endotoxins and (1–3)-β-d-glucans were collected from all sorting workers and loader operators during the work shift in both studied waste sorting plants. In total, 42 individual air samples were collected (29 in WSP1 and 13 in WSP2), which constituted a basis for assessment of workers exposure to dust. The samples were collected using individual aspirators Apex (Casella CEL Inc., USA), 25-mm-diameter glass fiber filters with 0.7 µm pore diameter (Whatman International Ltd., UK) at 2 l min^−1^ air flow during the work shift (samples with volumes ranging from 0.575 to 0.785 m^3^ were collected). Measuring sets (aspirator, tube and head with filter) were before each use calibrated with liquid calibrator Gilibrator 2 (Sensidyne, USA). In total, 42 samples were collected in both plants. From sampling to the time of the laboratory analysis, filters were stored frozen at −22°C.

Concentrations of inhalable dust were assessed by gravimetric method. Each filter was weighed in the laboratory before and after the measurements, using scales CP 225D (Sartorius, Germany) with an accuracy *d* of 0.01 mg.

### Elution of filters

To analyze endotoxins, the frozen filters were eluted in 10 ml of 0.05% Tween 20 (Cambrex, USA) solution in water free of endotoxins (Limulus Amebocyte Lysate water, Sigma, Germany). Samples were eluted on platform shaker for 15 min and then centrifuged at 1000×*g* for 15 min. From the resultant supernatant, 2090 µl of eluate was collected twice to be used for analysis of endotoxins and water-soluble fraction of (1–3)-β-d-glucans. In total, 10 M NaOH (Sigma, Germany) was added to the remaining supernatant so as to obtain the solution of 0.3 M NaOH concentration. Samples prepared like that were eluted on platform shaker for 10 min at 4 °C and then centrifuged at 1000xG for 15 min. The resultant supernatant was analyzed to check the presence of fraction of alkali-soluble (1–3)-β-d-glucans.

### Endotoxin analysis

Analysis of airborne endotoxins at workstations was based on the Polish Standard (PN-EN 14031:2006. The analysis was performed using the LAL (limulus amebocyte lysate) test in kinetic, chromogenic Kinetic-QCL version (Cambrex, USA). The eluate obtained earlier, using three consecutive dilutions method (1:5, 1:25, 1:125), was prepared to be placed on apyrogenic 96-well microplate for final analysis. Once the equal amounts (100 µl each) of the samples and LAL lysate were added, the concentrations of endotoxins were determined using the spectrophotometric reader SpectraMax Plus384 (Molecular Devices, USA) at 405 nm wavelength and the constant temperature of 37 °C. The results were obtained by comparing the samples with standard curve within the 0.049–100 EU ml^−1^ range, which was based on consecutive two-time dilutions of standard endotoxin (CSE) *Escherichia coli* 055:B5 with 15 EU ng^−1^ activity.

### (1–3)-β-d-Glucans analysis

Analysis of (1–3)-β-d-glucans was performed separately for both eluted [i.e., water-soluble (WS) and alkali-soluble (AS) fractions]. The determination was carried out using the Glucatell test in kinetic version (Associates of Cape Cod Inc., USA). Using the three consecutive dilutions method (1:5, 1:25, 1:125), the analyzed samples were prepared to be placed on 96-well microplate to perform the final analysis. Once 25 µl of samples and 50 µl of reagent were added to each well, the concentrations of (1–3)-β-d-glucans were determined using the spectrophotometric reader SpectraMax Plus384 (Molecular Devices, USA) with 405 and 490 nm wavelength light waves at the constant temperature of 37 °C. The results were obtained by comparing the samples with standard curve within the range 1.563–800 pg ml^−1^, which was based on consecutive two-time dilutions of standard (1–3)-β-d-glucan. The total concentration of (1–3)-β-d-glucans (respirable fraction) was the sum the total of the two (WS and AS) determined fractions. The values of the concentrations were expressed in ng m^−3^.

### Statistical analysis

The results were presented as median, geometric mean (GM), geometric standard deviation (GSD) and range (min–max). The Mann–Whitney *U* test was applied to detect statistically significant differences between the two waste sorting units, since the distributions were nonparametric. Spearman’s rank correlation coefficient was determined to analyze the association between the measured factors. For all calculations, a *p* value less than 0.05 was considered as significant. All calculations were obtained using STATISTICA v. 7.1 software package (Stat-Soft Poland).

## Results

The concentration of inhalable dust for the studied waste sorting plants ranged from 0.2 to 6.9 mg m^−3^, at the geometric mean of 1.3 mg m^−3^. Analysis of results indicated a significantly higher mean concentration of inhalable dust in WSP1, as compared to WSP2 (*p* < 0.05; 1.7 and 0.8 mg m^−3^, respectively). Pursuant to regulations mandatory in Poland, the value of the occupational exposure limit (OEL) for inhalable dust containing from 2 to 50% of free (crystalline) silica is 4 mg m^−3^ (The Ordinance of the Minister Labur and Social Policy of June 2014). Within the obtained results, this value was exceeded only in approx. 12% of workers (5 measurements, all related to WSP1).

The mean geometric concentration of endotoxins was 13.7 ng m^−3^, ranging from 1.6 to 78.9 ng m^−3^. No statistical significance was shown for differences in endotoxin concentrations between WSP1 and WSP2 (*p* > 0.05; 15.9 and 9.8 ng m^−3^, respectively).

The mean geometric concentration of (1–3)-β-d-glucans was 51.8 ng m^−3^ in the range 5.7–212.9 ng m^−3^. Similarly as in the case of endotoxins, no significant differences were found between the mean concentrations of this agent in the two studied plants (*p* > 0.05; 55.1 and 45.0 ng m^−3^ in WSP1 and WSP2, respectively). Comparison of the levels of water-soluble (WS) and alkali-soluble (AS) fractions of (1–3)-β-d-glucans did not found significant differences between WSP1 and WSP2.

**Fig. 1:**
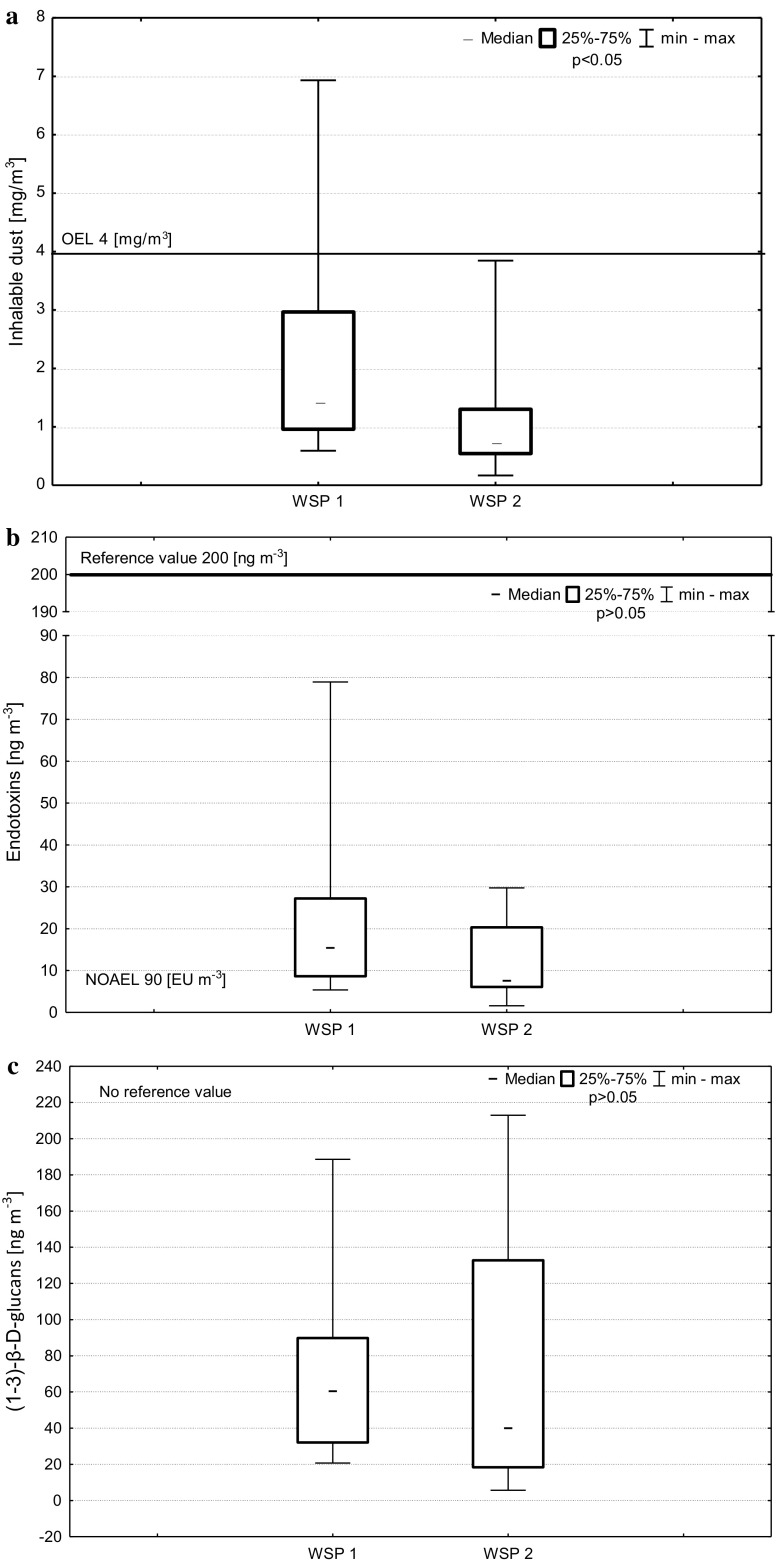
Concentrations of inhalable dust, endotoxins and (1–3)-β-d-glucans in studied WSPs (*N* = 42)

Table 2 and Fig. 1a–c show detailed results of assessment of the exposure of the workers of the municipal waste sorting plants to inhalable dust, endotoxins and (1–3)-β-d-glucans. The results indicate that in both plants most of the workers were exposed to inhalable dust in concentration below 2 mg m^−3^ (0.5 of OEL). In the case of endotoxins, it was indicated that in both plants a vast majority of workers performed their work in exposure to concentrations higher than NOAEL of 90 EU m^−3^. Having regard to the activity of standard endotoxin used for analyzing of studied air samples, NOAEL in our study was equal to a concentration of 6 ng m^−3^. But the maximum of concentrations of endotoxins did not reach even half of the reference value recommended for this agent in Poland (200 ng m^−3^) (Górny 2004).

**Table 2:** Concentrations of inhalable dust, endotoxins and (1–3)-β-d-glucans in studied waste sorting plants (*N* = 42)

Agent	WSP	Me	GM (GSD)	*p*	Range	
					Min	Max
Inhalable dust (mg m^−3^)	WSP1	1.43	1.66 (2.21)	**0.011**	0.59	6.93
	WSP2	0.74	0.84 (2.22)		0.17	3.84
	total	1.27	1.35 (2.26)	–	0.17	6.93
Endotoxins (ng m^−3^)^a^	WSP1	15.40	15.88 (2.08)	>0.05	5.36	78.94
	WSP2	7.60	9.81 (2.38)		1.58	29.75
	total	14.96	13.68 (2.22)	–	1.58	78.94
(1–3)-β-d-glucans (ng m^−3^)						
WS + AS	WSP1	60.37	55.13 (1.84)	>0.05	20.75	188.58
	WSP2	40.08	45.00 (3.17)		5.66	212.90
	Total	56.04	51.77 (2.24)	–	5.66	212.90
WS	WSP1	4.03	2.87 (1.70)	>0.05	1.08	9.82
	WSP2	6.92	2.34 (3.28)		0.29	11.09
	Total	4.20	2.70 (2.19)		0.29	11.09
AS	WSP1	73.39	52.26 (1.90)	>0.05	19.67	178.76
	WSP2	85.89	42.65 (2.90)		5.37	201.81
	Total	76.44	49.08 (2.31)		5.37	201.81

*GM* geometric mean, *GSD* geometric standard deviation, *Me* median

*p*—significance level difference between concentrations of harmful agents in WSP1 and WSP2 (for values in bold, *p* < 0.05)

WS—water-soluble fraction of (1–3)-β-d-glucans; AS—alkali-soluble fraction of (1–3)-β-d-glucans

^a^Standard endotoxin activity 15 EU ng^−1^ (value needed to convert endotoxin concentrations from ng m^−3^ to EU m^−3^)

No reference value for (1–3)-β-d-glucans has been set as yet.

**Fig. 2:**
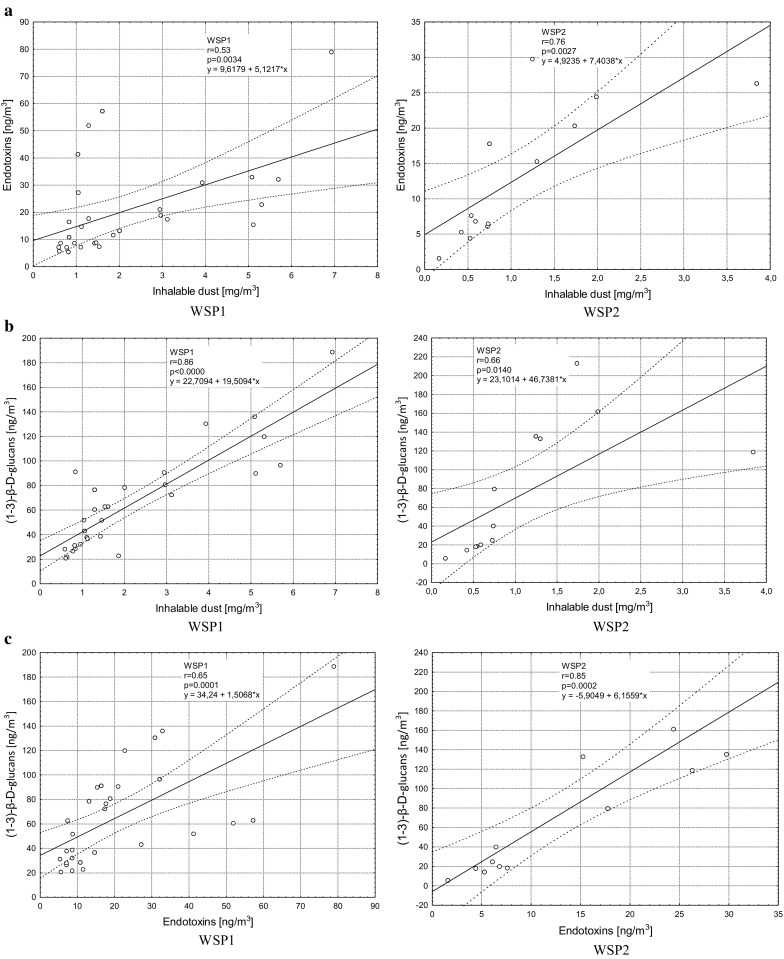
Correlations of inhalable dust, endotoxins and (1–3)-β-d-glucans in studied WSPs (*N* = 42)

Analysis of the results indicated significant (*p* < 0.05) and very high correlations between the concentrations of inhalable dust and endotoxins (*R* = 0.73), inhalable dust and (1–3)-β-d-glucans (*R* = 0.80) and endotoxins with (1–3)-β-d-glucans (*R* = 0.79) in both studied plants. All correlations are presented in Figs. 2(a),(b),(c).

## Discussion

Although the composition of dust sampled at workstations was not investigated, yet considering the morphology of wastes as its main source, and earlier findings in the literature (Dutkiewicz 1997; Gladding et al. 2003) we can assume that in plants of this type the dust of organic origin dominates. The low level of dust in the studied facilities was certainly due to efficient ventilation and air-conditioning systems in both sorting plants. As expected, a higher level of inhalable dust concentration was found in the plant of a higher processing capacity, which surely results from a much higher daily tonnage of the processed wastes. In the lower-capacity sorting plant, where a significantly lower concentration of dust was found, we should additionally note a high efficiency of natural ventilation through open doors for the trucks delivering wastes, which was important for air change in the relatively small working hall.

Sigsgaard et al. (1997) carried out in Denmark a study in waste sorting plants and found that the mean concentration of inhalable dust was at the level of 0.7 mg m^−3^, i.e., the level similar to the WSP2 plant in our study. Wouters et al. (2006) compared the results of the studies on occupational exposure among other to inhalable dust in workers of waste management plants, including presorting of residential waste. According to the results of individual measurements taken over several years, the average levels of exposure to this agent were determined for various occupational groups working in contact with wastes. And so it was determined that in the waste sorting plant the mean pollution with inhalable dust (GM) was 8.3 mg m^−3^ at the manual waste sorting station, 6.1 mg m^−3^ at the loader operator station, and 7.3 mg m^−3^ at the foreman’s station. These values are higher than the results obtained in our study. However, a comparison of our results with those published in another review study on the ranges of dust concentrations in waste sorting plants (Poulsen et al. 1995) showed that they correspond well with each other. Wouters et al. (2002) carried out a study on exposure to biological agents and their metabolites in a similar group of workers, i.e., waste collectors. The mean geometric value of inhalable dust concentration reached 0.6 mg m^−3^ at the range of 0.2–9.1 mg m^−3^. The results of the sorting plants which we examined correspond well with those obtained by Wouters et al. (2002). Although the mean geometric value for dust is a bit higher in our sorting plants, it is within the range of concentrations obtained by Wouters et al. (2002). Viegas et al. (2014) analyzed dust concentrations using a portable direct-reading type apparatus including fraction PM10 in two waste sorting plants in Portugal. The comparability of these results with our study is limited, because in our study also particles larger than 10 µm were retained on the filter. Therefore, the concentration of dust obtained in our study is by one order of magnitude higher than the concentration obtained in Portuguese waste sorting plants.

In our study, the mean level of endotoxin concentration for both plants in total reached 13.7 ng m^−3^ within the range from 0.6 to 78.9 ng m^−3^ (the corresponding values in terms of endotoxin units were 205.5 EU m^−3^; 23.7–1184.1 EU m^−3^). The reference value recommended in Poland for occupational environment is 200 ng m^−3^ or 2000 EU m^−3^ (Górny 2004); it was not exceeded in our study. There are also lower reference values; we should mention the standard of 25 ng m^−3^ devised by Laitinen (Laitinen et al. 2001) and 90 EU m^−3^ calculated as a weighed mean value for 8-hour working day, using the LAL test in kinetic version (DECOS 2010). Both values specified above were determined as NOAEL (no observed adverse effect level), i.e., the level at which no adverse health effects are observed in exposed people. In our study, these levels were exceeded, in former in almost one-fourth of the examined employees (10 samples >25 ng m^−3^), whereas in the latter almost in all employees (only five samples exhibited a level lower than 90 EU m^−3^).

Comparing the results of determinations of bacterial endotoxin concentrations in the waste sorting plants with the studies conducted by other authors in such plants, we should conclude that they are within a wide range of concentrations reported by other authors. In Denmark, in a study conducted among other in waste sorting plants, the mean concentration of endotoxins was found to reach 25 EU m^−3^, i.e., a level much lower than that found in our study (Sigsgaard et al. 1997).

Wouters et al. (2002), based on the results of individual measurements taken over several years, determined an average level of exposure to endotoxins for various occupational groups working in contact with wastes. It was determined that in the waste sorting plant, the mean pollution with endotoxins was 520 EU m^−3^ at the manual waste sorting station, 320 EU m^−3^ at the loader operator station and 29 EU m^−3^ at the foreman’s station. These mean values are somewhat higher than the mean values obtained in the presented study, but they are within the min–max range which we determined. In the Netherlands, a study was conducted on biological agents and their metabolites in workers who collect wastes. The geometric mean concentration of endotoxins was 39.4 EU m^−3^ at the range 4–7182 EU m^−3^. Exposure to biological agents in groups of workers collecting wastes as compared to those sorting wastes is similar (the same source of harmful biological agents), but usually the measured concentrations of respective agents, especially endotoxins, are somewhat higher in waste sorting plants because the work is performed indoors. The results of our studies correspond well with the Dutch study. The resultant geometric mean value is a bit higher but within the determined range of concentrations.

Hygienic interpretation of the results of analysis of (1–3)-β-d-glucan concentrations as an indicator of occupational exposure to fungi is difficult due to the lack of not only mandatory but even recommended reference levels of exposure. Such standards are missing in Poland and in other countries of the world. This is caused by gaps in the knowledge about the mechanism of specific effects on human organism (Schleibinger et al. 2004) and the lack of evidence of their exclusively harmful effects. The symptoms that appear in the upper airways due to exposure to fungi were found to result from inhalation of (1–3)-β-d-glucans and probably also other toxic compounds. It has been demonstrated experimentally that (1–3)-β-d-glucans interact with allergens and endotoxins (Douwes et al. 2003; Thorn et al. 2001). Pathological symptoms resulting from inhalation of (1–3)-β-d-glucans differ a bit from those observed in exposure to endotoxins (Rylander 2006); however, in complex exposure, these effects coincide with each other, and it is impossible to find out which agent is a direct cause (Liebers et al. 2008). The mean concentration of (1–3)-β-d-glucans in both waste sorting plants studied by ourselves was 51.8 ng m^−3^ in the range from 5.7 to 212.9 ng m^−3^. The relatively extensive range of concentrations of (1–3)-β-d-glucans is typical for this agent and should be considered not very high as compared to other studies conducted in different plants (Wouters et al. 2006). The concentrations of (1–3)-β-d-glucans that we indicated can hardly be compared to other results of studies because there are few articles of this type. Thorn et al. (1998) in a study conducted among workers collecting wastes found concentrations of (1–3)-β-d-glucans ranging from 10 to 36.4 ng m^−3^. These are values lower than those obtained by us in waste sorting plants, which could result from the fact that waste collection is conducted in open area (outdoors), as opposed to the sorting plants with indoor exposure. However, in the context of the high range of (1–3)-β-d-glucan concentrations quoted earlier, Wouters et al. (2006) recorded mean concentration of (1–3)-β-d-glucans of 1.22 µg m^−3^ level with the range 0.26–52.5 µg m^−3^ also for workers collecting wastes, although it was considerably more than in the sorting plants which we studied but Wouters et al. (2002) analyzed (1–3)-β-d-glucans by ELISA. Differences in obtained results can be caused by using different methods. The same authors in an earlier study, also on the waste collectors’ work environment, found out higher (than our results) mean concentration of (1–3)-β-d-glucans at the level of 1.29 µg m^−3^ (0.26–30.8 µg m^−3^). In two other studies related to occupational exposure of workers employed at waste management, the concentrations of approx. 40 ng m^−3^ were noted, i.e., similar to those obtained in the waste sorting plants which we studied (Gladding et al. 2003; Heldal et al. 2003).

Nielsen et al. (1997) published results of the study which investigated the relationships between different indicators of exposure to bioaerosol in occupational environment of waste collecting and recycling workers and categorized the quality of parameters for assessment of the exposure. The study demonstrates that the concentration of inhalable dust is one of the major indicators. The very high correlations shown in our study between the concentrations of inhalable dust with endotoxins and (1–3)-β-d-glucans constitute a hint for the choice of the most pertinent indicator for assessment of the level of worker exposure. With these results, we can assume that if there are high concentrations of inhalable dust of organic origin in the work environment, the concentrations of endotoxins and (1–3)-β-d-glucans will also be high. The measurement of the inhalable dust concentration is connected with much lower costs, as compared to the other agents discussed herein, which may be important for the employer who conducts a hygienic assessment of worker exposure at the workstations in municipal waste sorting plants.

To sum up the results of this study, we confirm the conclusions of other authors of studies on biological exposure in all types of waste management plants, including especially the waste sorting plants, that exposure to biological agents is unavoidable in that environment and constitutes a significant threat to workers’ health (Coccia et al. 2010; Douwes et al. 2003; Viegas et al. 2015; Wouters et al. 2006). However, it is possible to decrease the health effects in exposed workers through implementation of an appropriate preventive program based on separation of the source of harmful agents from the worker by employing airtight and automatic working process, minimizing dust content in the ambient air proper ventilation of workstations, equipping the workers with correctly chosen individual protection means, especially those protecting the respiratory tract, and by systematic and detailed training courses for all exposed workers on the protection against harmful biological agents.

## Conclusions

Although a low number of samples constitute some limitation, the results lead to the following conclusions:

1. Processing capacity of waste sorting plants differentially affects concentrations of inhalable dust, whereas concentrations of endotoxins and glucans are less clearly affected. This suggests that relative concentrations of endotoxin and glucan are depending on the waste sorting capacity.
2. Significant and very high correlations were found between concentrations of inhalable dust, endotoxins and airborne (1–3)-β-d-glucans at workstations in municipal waste sorting plants, regardless of the plants’ capacity.
3. Results of the study point to the need for further research on pathological symptoms associated with exposure to inhalable dust, endotoxins and (1–3)-β-d-glucans in the municipal waste sorting plant environment.
